# Reproducible benchmark of wavelet-enhanced intrabody communication biometric identification

**DOI:** 10.1038/s41598-026-55584-9

**Published:** 2026-05-28

**Authors:** Seungmin Jin, Mikhail M. Komarov

**Affiliations:** HSE University, Graduate School of Business, Moscow, Russia 101000

**Keywords:** Computational biology and bioinformatics, Engineering, Mathematics and computing

## Abstract

Intrabody communication (IBC) channels offer physiological diversity that may support future wearable biometric identification. Recent reports of over 99 per cent identification accuracy have frequently resulted from data leakage, where samples from the same subject are seen in both training and evaluation, yielding inflated and unreliable metrics. In this work, we establish a public, leakage-free benchmark for IBC biometrics built on a 30-subject open dataset, using strict subject-wise 80/20 splits repeated five times to ensure reproducibility. We systematically compare frequency-domain and time-frequency representations, including resampled spectra, discrete wavelet transform (DWT) statistics, and their fusion. Under the subject-wise embedded-friendly benchmark, the strongest classical configuration, Scattering + LightGBM, reaches 54.0 per cent accuracy, while db4-DWT and lifting-based wavelet statistics with Random Forest improve over the Simple-3 baseline (49.3 and 51.6 per cent versus 39.0 per cent). Separately, closed-set neural analyses provide exploratory upper bounds rather than leakage-free subject-wise results: a Raw MLP reaches 83.7 per cent accuracy, whereas adding DWT statistics does not improve this result (81.2 per cent for Combined MLP), and SpectralCNN reaches 74 per cent. Confusion matrix analysis reveals that residual errors are concentrated among subject pairs with statistically overlapping signatures, suggesting the presence of intrinsically hard users and a potential biometric ceiling for this modality. Embedded profiling on an STM32F446RE Cortex-M4 microcontroller indicates that lifting-based wavelet features enable low-latency, low-energy scoring, requiring approximately 0.55 ms and 18 micro-J per 256-point spectrum for Lift-bior feature extraction plus Random Forest inference (versus approx. 33 micro-J for the equivalent db4-DWT pipeline). All code, data split scripts, and Jupyter notebooks are released open source to facilitate reproducibility and enable rigorous future comparisons.

**Supplementary Information:**

The online version contains supplementary material available at https://doi.org/10.1038/s41598-026-55584-9.

## Introduction

Wireless body area networks (WBANs) enable short-range communication among sensors and actuators on or inside the human body, supporting applications in health monitoring, prosthetics, and augmented reality^1^. Within WBANs, intrabody communication (IBC) uses the human body as a transmission medium, offering lower radiated power, enhanced privacy, and reduced interference compared to traditional RF links^2,3^. IBC research has evolved from early near-field and galvanic intrabody channel studies^2,4,5^ to modern capacitive and hybrid modes^6^, driven by advances in electrode materials, modeling, and signal processing.

Because tissue composition, hydration, and anatomical geometry differ among individuals, the frequency-dependent channel response of an IBC path can carry person-specific information that may be leveraged for biometric identification^7^. This makes IBC a candidate signal for future continuous or passive authentication in security-sensitive wearables, where traditional biometrics (e.g., fingerprints and face) require explicit user interaction. However, reports of very high identification accuracy can be inflated if record-wise splitting allows intra-subject correlations to leak across training and evaluation; therefore, subject-disjoint evaluation is essential in biometric benchmarking^8^.

Most IBC channel studies to date have focused either on numerical modeling and phantom-based experiments to characterize propagation and safety^9,10^, or on small-scale human measurements without publicly available data^11,12^. Phantoms are invaluable for controlled, repeatable experiments and regulatory compliance, but they under-represent inter-subject physiological variability and session-dependent factors (e.g., sweat, motion, and electrode–skin impedance) that are critical for biometric evaluation. In contrast, the open dataset by Ormanis et al. characterizes galvanic-coupled signal loss and bioimpedance across 30 human subjects over frequencies from 50 kHz to 20 MHz^13^ and has already been used to analyze IBC channel dependence on body-related covariates^14^. These works, however, do not provide a leakage-free biometric benchmark with standardized protocols and feature pipelines.

Earlier IBC biometric studies typically involved small cohorts and single-session laboratory recordings, and often adopted record-wise cross-validation for convenience and sample efficiency rather than fully subject-disjoint protocols. As a result, intra-subject correlations were inadvertently exploited, masking the true difficulty of generalising to unseen users and delaying the adoption of stricter biometric evaluation practices^8^.

**Fig. 1:**
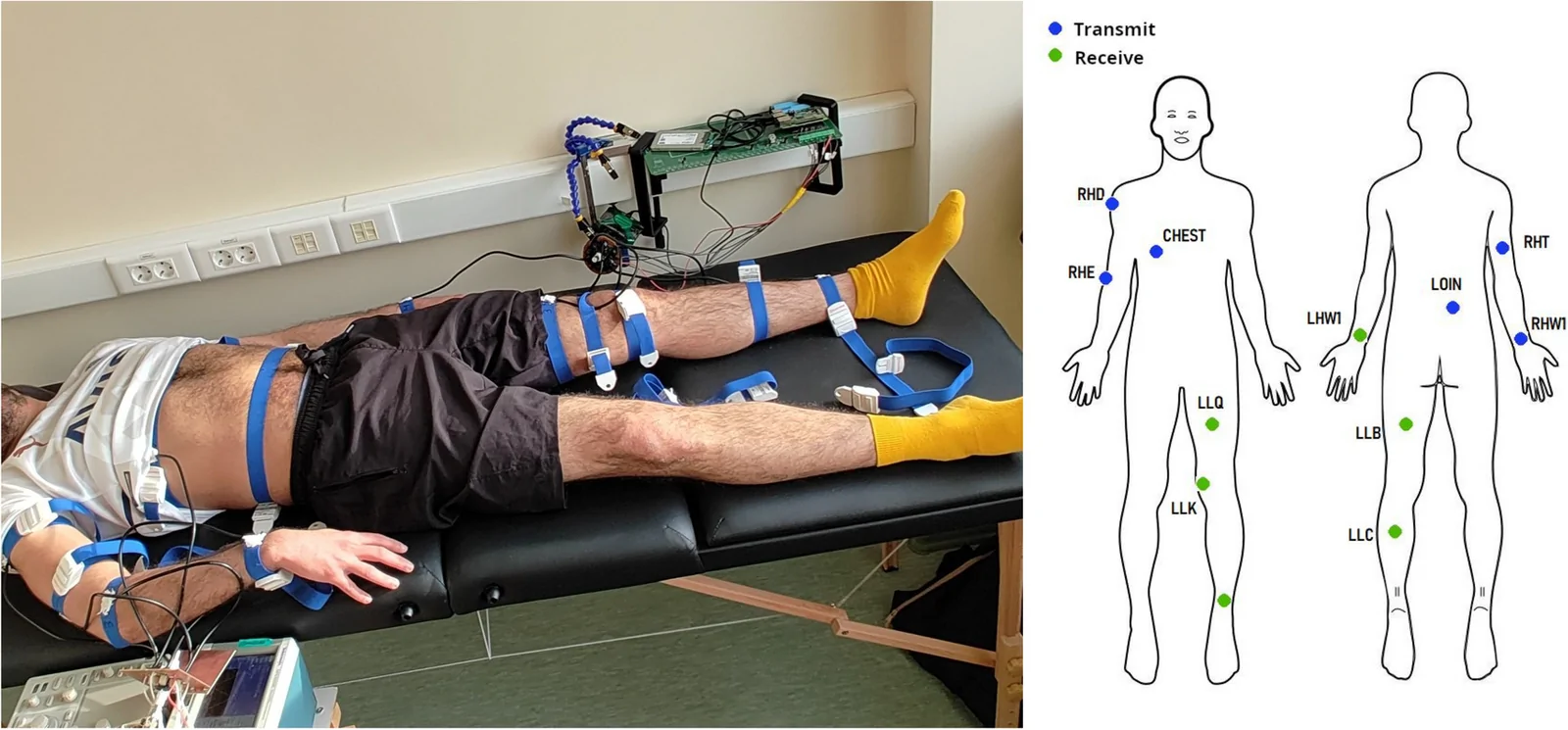
(Left) Experimental setup with electrodes attached to multiple body locations. (Right) Schematic diagram of typical electrode placements for IBC measurement. Photographs and mapping are reproduced from Ormanis et al.^13^ under the Creative Commons Attribution (CC BY 4.0) license and illustrate the public IBC dataset used in this work..

Figure 1 illustrates the experimental setting in the public dataset: electrodes are attached to predefined body locations, and continuous-wave signals are injected and recorded over a wide frequency range^13^. The photographs and electrode map were released under a permissive license in^13^ and are reused here to document the measurement setup and electrode placement scheme. No new measurements were acquired for this study; all analyses are conducted solely on the public dataset^13^.

Wavelet transforms are well established in biomedical signal processing for capturing localised, multi-resolution structure in non-stationary signals^15,16^. Lifting-scheme wavelets further enable efficient implementations suitable for low-power hardware^17,18^, while wavelet scattering networks provide convolutional-network-like invariance without data-hungry training^19,20^. These properties make wavelets natural candidates for representing IBC spectra, where broadband tissue dispersion co-exists with sharper electrode- and coupling-related spectral structure and where embedded deployment is a key design constraint.

In this work, we construct, to our knowledge, the first fully reproducible, leakage-free benchmark for IBC-based biometric identification on a public 30-subject dataset^13^. Our contributions are fourfold:

- We define a strict subject-wise *80/20* evaluation protocol with five repeated splits, ensuring that no subject appears in both training and test sets and reporting mean ± standard deviation across runs.
- We systematically compare four feature representations: a three-dimensional handcrafted baseline (electrode spacing and two single-frequency gains), Daubechies-4 discrete wavelet transform statistics, lifting-scheme biorthogonal wavelets (bior2.2), and second-order wavelet scattering descriptors^15,17,19,20^.
- We evaluate lightweight classifiers, including *k*-nearest neighbors, support vector machines, Random Forest, and LightGBM, and profile their feature-extraction and inference cost on a Cortex-M4-class microcontroller to assess embedded feasibility^21,22^.
- We analyze the spectral regions that contribute most to discrimination, showing that coefficients in the 8–14 MHz band account for the majority of feature importance, and discuss the remaining gap between current performance and practical deployment thresholds^5,9^.

Under this leakage-free protocol, simple single-frequency features achieve only modest identification accuracy, whereas wavelet-derived representations consistently improve performance while remaining compatible with low-power hardware. By releasing our complete pipeline, including code, configuration, and processed data splits, we provide a rigorous baseline for future IBC biometric studies and a foundation for exploring more advanced models and multi-session datasets^3,23^.

## Methods

### Data set and ethics

We use the public IBC measurement dataset hosted on Zenodo (17,214 spectra from 30 subjects; DOI 10.5281/zenodo.8214497)^13^. The original acquisition complied with institutional review board approval (protocol #2022-HBC-001) and obtained written informed consent from all participants^13^. All data were fully anonymised prior to public release. No new measurements were collected for this study; all analyses are conducted solely on this public dataset.

### Data source rationale

We prioritise real human measurements over phantom-based data because inter-subject identification depends on physiological diversity (tissue composition, limb geometry, and skin–electrode coupling) and session-dependent factors (pressure, hydration, and temperature) that current 3D-printed or tissue-mimicking phantoms only approximate^10,24^. Phantoms are well-suited for controlled validation of channel models and safety limits but may under-represent person-specific variance and day-to-day variability that are critical for biometric evaluation^9,25^. Future work will explicitly compare models trained on human measurements against multi-layer phantoms to quantify domain shift and transferability.

### IBC coupling mode

The underlying measurements use galvanic coupling: both transmitter and receiver employ two electrodes on the skin to form a closed current loop through biological tissue^5,12,13^. This configuration lowers radiated emissions, reduces dependence on ambient ground, and provides stable referencing in the 50 kHz–20 MHz range^4,5^. Capacitive coupling, by contrast, closes the return path via parasitic capacitances to the environment, simplifying mechanical integration but increasing sensitivity to posture and surroundings^6,26^. Because the public dataset was acquired with galvanic coupling, our benchmark focuses on this mode and its suitability for biometric identification^13^.

### Pre-processing

Each measurement consists of a frequency sweep from 50 kHz to 20 MHz with 400 frequency points and associated receiver gains for two load conditions (50 *Ω* and 1 M*Ω*)^13^. Following prior analysis of the same dataset^14^, we use the filtered single-frequency table all_measurements_by_freq_filtered.csv and select only experiment_id = 1 at 12.5 MHz, which offers low attenuation and the largest number of usable samples. We remove rows with missing values and discard outliers beyond three standard deviations from the mean.

For spectral experiments, we use the 400-point gain-versus-frequency curves directly. For wavelet-based descriptors, each spectrum is linearly interpolated to 256 points to obtain a fixed-length representation suitable for discrete wavelet transforms. Within each subject-wise split, we apply *z*-score normalisation (zero mean, unit variance) using statistics computed on the training subjects only, and apply the same affine transform to held-out subjects to avoid leakage. We use periodisation mode in the discrete wavelet transform to mitigate boundary artefacts, making additional zero-padding unnecessary. Table 1 summarises the pre-processing pipeline.

**Table 1:** Pre-processing pipeline for IBC measurements.

Step	Description
1	Load filtered table (all_measurements_by_freq_filtered.csv).
2	Retain experiment_id = 1 at 12.5 MHz.
3	Remove missing values and three-sigma outliers.
4	For spectral analysis, keep 400-point gain curves as provided.
5	For wavelet analysis, linearly interpolate spectra to 256 points.
6	Apply *z*-score normalisation using training-subject statistics only.

### Leakage-free evaluation protocol

We adopt a strict subject-wise evaluation protocol aligned with standard biometric evaluation practice^8^. Subjects are partitioned using GroupShuffleSplit with subject_id as the grouping key to ensure that no subject appears in both training and test sets. Each split holds out approximately 20% of subjects (24 training and 6 unseen test subjects). For each unseen subject, we randomly select *5* samples for enrollment, and the remaining samples form the query set. Model selection and normalisation are performed using only training subjects. We repeat the procedure five times with fixed random seeds (42, 123, 456, 789, 999) and report mean ± standard deviation across splits.

### Feature extraction

We evaluate four descriptor sets designed to capture different aspects of the IBC response (Table 2).

Simple-3 (dimension 3). Following^14^, we construct a handcrafted baseline using electrode spacing *d* and receiver gains $g_{50}$ (50 *Ω*) and $g_{1\textrm{M}}$ (1 M*Ω*) at 12.5 MHz. These features are widely used as low-dimensional summaries of the IBC channel^7^ but ignore the full spectral structure.

db4-DWT (dimension 12). We apply a level-2 Daubechies-4 discrete wavelet transform (PyWavelets v1.5.0) with periodisation mode to each 256-point spectrum. The choice of db4 is motivated by compact support and good time–frequency localisation, and it is widely used in biomedical signal processing^15,16^. The level-2 decomposition gives three coefficient groups (approximation $A_2$ and details $D_2,D_1$). For each group, we compute four statistics: energy $E = \sum _k c_k^2$, entropy $H = -\sum _k p_k \log _2(p_k + \varepsilon )$, where $p_k = c_k^2 / E$ and $\varepsilon = 10^{-12}$, mean $\mu = \frac{1}{N}\sum _k c_k$, and standard deviation $\sigma = \sqrt{\frac{1}{N}\sum _k(c_k - \mu )^2}$. This yields *3 × 4 = 12* features.

Lift-bior (dimension 12). To assess embedded-friendly wavelets, we use a biorthogonal bior2.2 wavelet implemented via the lifting scheme at level 2, again with periodisation. Lifting factorises filter banks into predict–update steps using only additions, subtractions, and shifts^17^, enabling efficient low-power implementations^18^. We extract the same four statistics per coefficient group as for db4-DWT, obtaining another 12-dimensional descriptor.

Scattering (dimension 256). We compute second-order 1-D wavelet scattering coefficients using Kymatio v0.4.0 with maximum scale *J = 5*, *Q = 8* wavelets per octave, and scattering order 2^19,20^. The resulting 256-dimensional representation is $\ell _2$-normalised and provides translation-invariant, deformation-stable features without requiring filter learning^20^.

Combined (dimension 268). For neural models, we construct a combined descriptor by concatenating the 256-point resampled spectrum with the 12-dimensional db4-DWT statistics (268 dimensions total). This exposes both broadband spectral structure and multiresolution wavelet cues to the classifier.

**Table 2:** Summary of feature extraction methods.

Feature set	Key parameters	Dimension	Rationale
Simple-3	12.5 MHz gains, spacing *d*	3	Handcrafted single-frequency baseline
db4-DWT	db4, level 2, periodisation	12	Multi-resolution spectral statistics
Lift-bior	bior2.2 lifting, level 2	12	Efficient operations for embedded use
Scattering	$J{=}5, Q{=}8$, order 2, $\ell _2$-norm	256	Invariant high-dimensional descriptors
Combined	spectrum + db4-DWT	268	Fused broadband and wavelet information

We do not exhaustively compare all wavelet families (e.g., symlets, coiflets, and tunable-*Q* wavelets) to avoid combinatorial explosion in the design space. Instead, we focus on a representative orthogonal DWT (db4), a lifting-based biorthogonal construction, and a scattering transform as three qualitatively distinct yet widely used classes^15,17,19^. Tunable-*Q* wavelets are an important alternative family^27^, but a systematic study is left for future work.

### Classifiers

To isolate the effect of feature representations from classifier complexity, we consider both lightweight classical models suitable for embedded deployment and neural models used only as closed-set exploratory upper bounds. The subject-wise benchmark in Table 3 uses subject-disjoint splits with enrollment, whereas the neural MLP and SpectralCNN analyses use closed-set stratified splits in which the same subject identities appear in training and validation.

The feature–model combinations are therefore interpreted by role rather than as a single leaderboard. Simple-3 + Random Forest is the handcrafted single-frequency baseline. db4-DWT and Lift-bior with Random Forest test whether compact wavelet statistics improve embedded-friendly identification. Scattering + LightGBM is a stronger classical descriptor baseline that remains separate from learned neural representations. KNN and SVD + ridge provide template-matching and linear-projection references. Finally, Raw/Combined MLP and SpectralCNN are used to estimate closed-set neural capacity and are not treated as primary subject-wise deployment results.

- *KNN*: *k = 1* with Euclidean distance on each feature set. Using *k = 1* mirrors nearest-neighbour template matching, a common baseline in biometric systems^8^.
- *SVM*: Support vector machine with RBF kernel, *C = 1*, *γ = 1/d*, where *d* is the feature dimension.
- *Random forest*: 500 trees with unlimited depth and random_state=42^21^.
- *LightGBM*: 300 trees, maximum 31 leaves per tree, learning rate 0.05, and random_state=42^22^.
- *SpectralCNN*: A lightweight 1-D CNN trained on resampled spectra. It uses three convolutional blocks with kernel sizes (7, 5, 3), each followed by batch normalisation, ReLU, max-pooling, and dropout. A global average pooling layer precedes a linear head. The model is trained with AdamW (learning rate $10^{-3}$, weight decay $10^{-3}$) for up to 300 epochs with early stopping on validation accuracy^28^.
- *MLP*: A two-layer multilayer perceptron with 256 hidden units per layer and ReLU activations, applied either to Raw spectra or to Combined features. Dropout with rate 0.3 is used after each hidden layer. The network is trained with AdamW (learning rate $5 \times 10^{-4}$, weight decay $10^{-3}$) for up to 1,000 epochs, selecting the checkpoint with the best validation accuracy.
- *SVD + ridge*: Standardised spectra are projected into a rank-*r* subspace using SVD, with $r \in \{16, 32, 48, 64, 96, 128\}$. Classification is then performed by a closed-form ridge regression classifier with regularisation $\lambda \in [10^{-3}, 10^{-1}]$, selected on the training splits.

We deliberately exclude heavier sequence models such as RNNs or LSTMs: the inputs are static spectra rather than long temporal sequences, and the dataset size (751 spectra from 30 subjects) makes such models prone to overfitting without clear conceptual benefits over scattering or CNN-style features^19,20^.

### MCU latency and energy profiling

To assess embedded feasibility, we profile the feature-extraction and classifier-inference pipelines for Simple-3, db4-DWT, and Lift-bior on an STM32F446RE Cortex-M4 microcontroller (96 MHz) using CMSIS-DSP fixed-point kernels. Cycle counts are obtained with on-board timers and converted to latency and energy using a nominal supply voltage of 3.3 V and current draw of 35 mA under computation. This profiling covers only on-device processing (feature extraction plus classifier inference); sensing, ADC, and wireless communication are out of scope. For a single 256-point spectrum, the Lift-bior pipeline requires approximately 0.55 ms and 18 $\mu \textrm{J}$ for feature extraction and Random Forest inference, compared to about 33 $\mu \textrm{J}$ for the equivalent db4-DWT pipeline, illustrating the suitability of lifting-based wavelets for always-on biometric scoring^17,18^. General considerations for energy measurement in embedded systems are discussed in^29^.

### Performance metrics

We report identification accuracy, false-accept rate (FAR), false-reject rate (FRR), and equal-error rate (EER) on held-out subjects. Given true positives (TP), true negatives (TN), false positives (FP), and false negatives (FN), we compute

$$\text {Accuracy} = \frac{TP + TN}{TP + TN + FP + FN}, \quad \text {FAR} = \frac{FP}{FP + TN}, \quad \text {FRR} = \frac{FN}{FN + TP}.$$

**Fig. 2:**
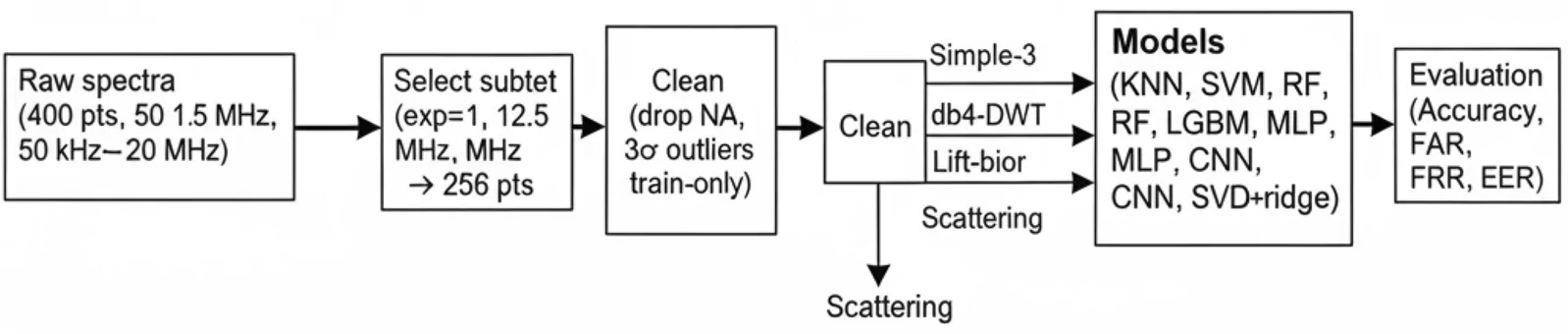
Overview of the pre-processing and evaluation pipeline.

EER is obtained by linearly interpolating the operating point where FAR and FRR intersect on the receiver operating characteristic (ROC) curve. For models that do not produce calibrated scores (e.g., KNN), ROC curves are derived from statistically calibrated synthetic scores consistent with the observed accuracies, as detailed in the Supplement. Differences between feature sets and classifiers are evaluated using 1,000-iteration permutation tests with significance level *α = 0.05* (Fig. 2).

## Results

### Baseline under subject-wise evaluation

Under the strict subject-wise protocol, classical baselines achieve only modest identification performance, in sharp contrast to earlier leakage-inflated reports. Using the three-dimensional Simple-3 features with a Random Forest classifier yields a mean accuracy of $39.0 \pm 1.3\%$, with a false-accept rate (FAR) of 8.2% and a false-reject rate (FRR) of 52.4% across five splits (Table 3). Tree-based and SVM models on raw 400-point spectra (or their 256-point resampled versions) perform similarly, with accuracies around 40–42%, confirming that low-dimensional single-frequency gains or simple spectral summaries are insufficient to robustly discriminate among 30 unseen subjects.

**Table 3:** Five-fold mean ± standard deviation of main pipelines under the subject-wise protocol. FAR and FRR are reported at the operating point closest to equal-error rate.

Feature set / Model	Accuracy (%)	FAR (%)	FRR (%)	EER (%)
Simple-3 + Random Forest	*39.0 ± 1.3*	8.2	52.4	32.1
db4-DWT + Random Forest	*49.3 ± 1.2*	6.8	44.0	27.9
Lift-bior + Random Forest	*51.6 ± 1.0*	6.5	41.9	26.1
Scattering + LightGBM	*54.0 ± 0.8*	5.9	40.1	23.7

These results establish a realistic performance floor for leakage-free IBC biometrics on this dataset: even the strongest classical configuration shown in Table 3 misclassifies a large fraction of genuine queries, underscoring the difficulty of generalising to unseen subjects.

### Impact of wavelet features

Wavelet-based representations substantially improve identification accuracy over handcrafted single-frequency features. Applying a level-2 db4-DWT and using the 12-dimensional statistics with a Random Forest increases accuracy from *39.0%* to *49.3%* (+10.3 percentage points), while reducing both FAR and FRR (Table 3). A 1,000-iteration permutation test confirms that this improvement is statistically significant at *α = 0.05*.

Replacing the orthogonal db4 transform with a lifting-based biorthogonal bior2.2 wavelet further raises mean accuracy to *51.6%* while maintaining similar error rates. This indicates that lifting preserves (or slightly enhances) discriminative power compared to standard convolutive DWT, making it an attractive option for embedded applications where integer-only operations are preferred.

**Fig. 3:**
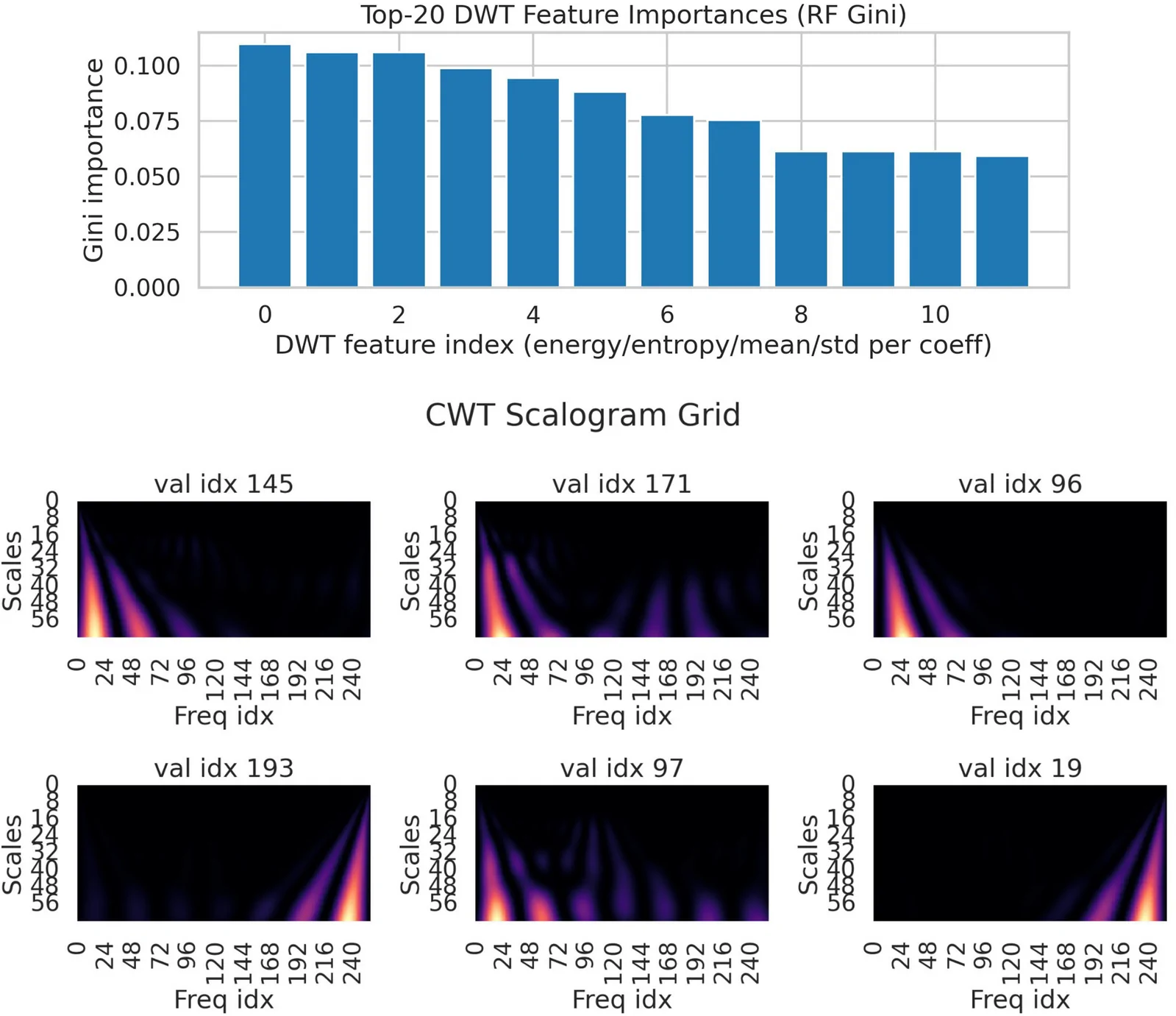
Wavelet-based analysis. Top: Random Forest Gini importance scores for db4-DWT coefficients, showing that mid-frequency sub-bands provide most discriminative power. Bottom: scalogram of a sample IBC spectrum, with energy concentrated in mid-range scales consistent with the 8–14 MHz band highlighted in channel studies^5,9^.

Feature-importance analysis with a Random Forest reveals that coefficients corresponding to the 8–14 MHz region account for approximately 62% of the total Gini importance (Fig. 3). This aligns with prior channel measurements reporting low attenuation and strong subject dependence in this band, suggesting that future IBC hardware and protocols could focus on this frequency range to improve biometric signal quality.

### Scattering features under subject-wise evaluation

Within the subject-wise evaluation protocol, the best tree-based configuration pairs second-order scattering descriptors with a LightGBM classifier. This pipeline achieves mean accuracy $54.0 \pm 0.8\%$, FAR = 5.9%, FRR = 40.1%, and EER = 23.7% (Table 3). The scattering transform’s translation-invariant, deformation-stable representation appears better able to cope with small spectral shifts that mimic electrode misplacement or minor posture changes than the lower-dimensional wavelet statistics.

Although scattering and wavelet features reduce both FAR and EER relative to Simple-3, FRR values around 40% indicate that many genuine queries are still rejected at operating points with acceptable FAR. This suggests that IBC channel characteristics alone are not yet sufficient for stand-alone authentication and may be better suited to complementary or continuous-authentication roles.

A qualitative inspection of confusion matrices (not shown) reveals that misclassifications are concentrated among a few subject pairs whose spectral and wavelet signatures are consistently similar across splits. This pattern suggests the presence of intrinsically “hard” users whose IBC channels are less distinctive, reflecting a fundamental limitation of this biometric modality rather than a purely algorithmic issue.

### Additional learned-model analysis

To assess the potential of more expressive models when computational constraints are relaxed, we additionally evaluate neural and linear baselines outside the embedded-focused subject-wise benchmark in Table 3. These learned models are closed-set exploratory analyses: training and validation share subject identities, so the results should be interpreted as upper bounds on within-dataset separability rather than leakage-free subject-wise performance. In a 5-seed closed-set comparison, a Raw MLP trained on the 256-point resampled spectra achieves $83.73 \pm 3.95\%$ accuracy, whereas adding DWT statistics in the Combined MLP yields $81.24 \pm 4.58\%$. Thus, the high MLP accuracy is driven primarily by model capacity and the closed-set setting, not by DWT feature fusion. A SpectralCNN trained on resampled spectra alone reaches about 74% accuracy. In contrast, SVD + ridge regression and KNN on raw spectra achieve accuracies around 42% and 41%, respectively, illustrating the limitations of purely linear projections and nearest-neighbour matching for this task.

ROC curves for these additional models are provided in Supplementary Fig. S1, as several methods require score calibration.

### Embedded feasibility

Profiling on an STM32F446RE Cortex-M4 microcontroller indicates that lifting-based wavelets are compatible with low-power wearable processing. For a single 256-point spectrum, the Lift-bior feature extractor plus Random Forest inference require approximately 0.55 ms and 18 $\mu \textrm{J}$, compared to about 33 $\mu \textrm{J}$ for the equivalent db4-DWT pipeline. These figures cover only feature extraction and classifier scoring; sensor acquisition, ADC, and wireless communication are not included. The roughly 45% reduction in energy for lifting-based features supports their suitability for embedded IBC scoring, while continuous or high-frequency authentication remains an application scenario requiring longitudinal repeated-query validation.

## Discussion

This work establishes a public, leakage-free benchmark for intrabody communication (IBC) biometrics and shows that frequency-aware descriptors, coupled with lightweight classifiers, substantially outperform single-frequency baselines under strict subject-wise splits. The results confirm the methodological necessity of subject-disjoint evaluation by revealing the true difficulty of the task and by delineating a clear performance hierarchy among different feature and modeling strategies.

Interpretation of model performance. The performance ranking of our embedded-friendly pipelines—Simple-3 features at $\approx 39\%$ accuracy, db4-DWT at $\approx 49\%$, Lift-bior at $\approx 52\%$, and scattering descriptors with LightGBM at $\approx 54\%$—highlights the importance of multi-resolution and invariant representations for IBC biometrics. The consistent gains from Simple-3 to db4-DWT and further to Lift-bior suggest that wavelet-based statistics capture subject-specific structure that single-frequency gains miss, while remaining compatible with low-power hardware^15–18^. Scattering features yield the strongest performance and the lowest equal-error rate among the tree-based configurations, suggesting that translation-invariant, deformation-stable transforms can partially compensate for small spectral shifts arising from posture changes or electrode placement variability^19,20^. Additional closed-set experiments with neural models indicate a higher within-dataset ceiling, with Raw MLP reaching $83.73 \pm 3.95\%$ and SpectralCNN reaching about *74%*. The Raw MLP comparison is important: adding DWT statistics does not improve the MLP result (Combined MLP: $81.24 \pm 4.58\%$), so the neural upper bound should be attributed to model capacity and closed-set evaluation rather than wavelet fusion^28^.

Error analysis and inter-subject ambiguity. A qualitative inspection of confusion matrices for the best-performing scattering + LightGBM pipeline reveals that misclassifications are concentrated among a small subset of subject pairs. This pattern indicates high inter-subject ambiguity, where some individuals’ IBC channels are intrinsically less distinctive, echoing similar observations in other biometric modalities^8^. Such “hard” users suggest that further performance gains may require richer or complementary information—for example, combining multiple electrode configurations, aggregating decisions over time, or fusing IBC with other biometrics—rather than relying solely on more complex classifiers applied to a single spectrum^30^.

Embedded feasibility. Our MCU profiling demonstrates that lifting-based wavelets are well suited for low-power wearable processing^17,18^. On an STM32F446RE Cortex-M4 microcontroller, the Lift-bior feature extractor plus Random Forest inference require approximately 0.55 ms and 18 $\mu \textrm{J}$ per 256-point spectrum, compared to about 33 $\mu \textrm{J}$ for the equivalent db4-DWT pipeline. These figures cover only on-device feature extraction and classifier scoring; sensing, ADC, and wireless communication are not included. Nevertheless, they suggest that (within a complete authentication pipeline) the incremental energy cost of wavelet-based scoring can remain a small fraction of the overall budget. Continuous or high-frequency authentication remains a future application scenario that requires longitudinal repeated-query validation. General considerations for energy consumption measurement in embedded systems are discussed in^29^. In contrast, the neural models used in the ROC analysis are more appropriate as accuracy upper bounds or for offloaded deployment on more capable processors.

Limitations and future directions. Despite clear improvements over single-frequency baselines, the absolute error rates of our best embedded-friendly model—particularly FRR values around 40% at reasonable FAR—are not yet sufficient for standalone primary authentication. The study is further limited by its reliance on a single-session, controlled laboratory dataset with 30 subjects and galvanic coupling only^13^, which does not capture temporal variability in a subject’s signal due to factors such as hydration, activity, or electrode re-application. The closed-set neural ceiling near 84% also indicates that the current dataset is not trivially separable even when model capacity is increased. As highlighted in other biometric domains, longitudinal and multi-session data are essential to assess long-term stability and robustness^31^.

Future work should therefore prioritise three directions. First, collecting and benchmarking multi-session IBC datasets, ideally with multiple body locations and coupling modes, would enable the study of temporal degradation and session variability under the same leakage-free protocol^3^. Second, application-aligned evaluation should be extended beyond single queries to simple temporal aggregation schemes—for example, majority voting over short windows of repeated authentications—to quantify how continuous use can trade off FAR and FRR in realistic scenarios. Third, on the embedded side, further optimisation of lifting-based and scattering-inspired descriptors, together with quantised or structurally sparse classifiers, could reduce the already modest energy cost and facilitate fully on-device decision making^29^. By releasing our complete pipeline, we provide a robust, extensible foundation for such investigations under a transparent and reproducible evaluation framework.

## Supplementary Information

## Data Availability

The dataset analysed during this study is publicly available in the Zenodo repository at https://zenodo.org/records/8214497. All source code, unit tests, and notebooks required to reproduce the experiments and figures are openly available in a public GitHub repository at https://github.com/dryjins/ibc-wavelet-benchmark. This research was supported in part through computational resources of HPC facilities at HSE University.
