# Supplementary material for: Reproducible benchmark of wavelet-enhanced intrabody communication biometric identification
Source: Sci Rep. 2026 May 28;16:24421. doi: 10.1038/s41598-026-55584-9 (PMC13448553; doi:10.1038/s41598-026-55584-9)
Supplement: Supplementary file 1 [file 41598_2026_55584_MOESM1_ESM.pdf]

Supplementary Information

Reproducible Benchmark of Wavelet-Enhanced Intrabody Communication Biometric Identification

Seungmin Jin, Mikhail M. Komarov

Supplementary Figures

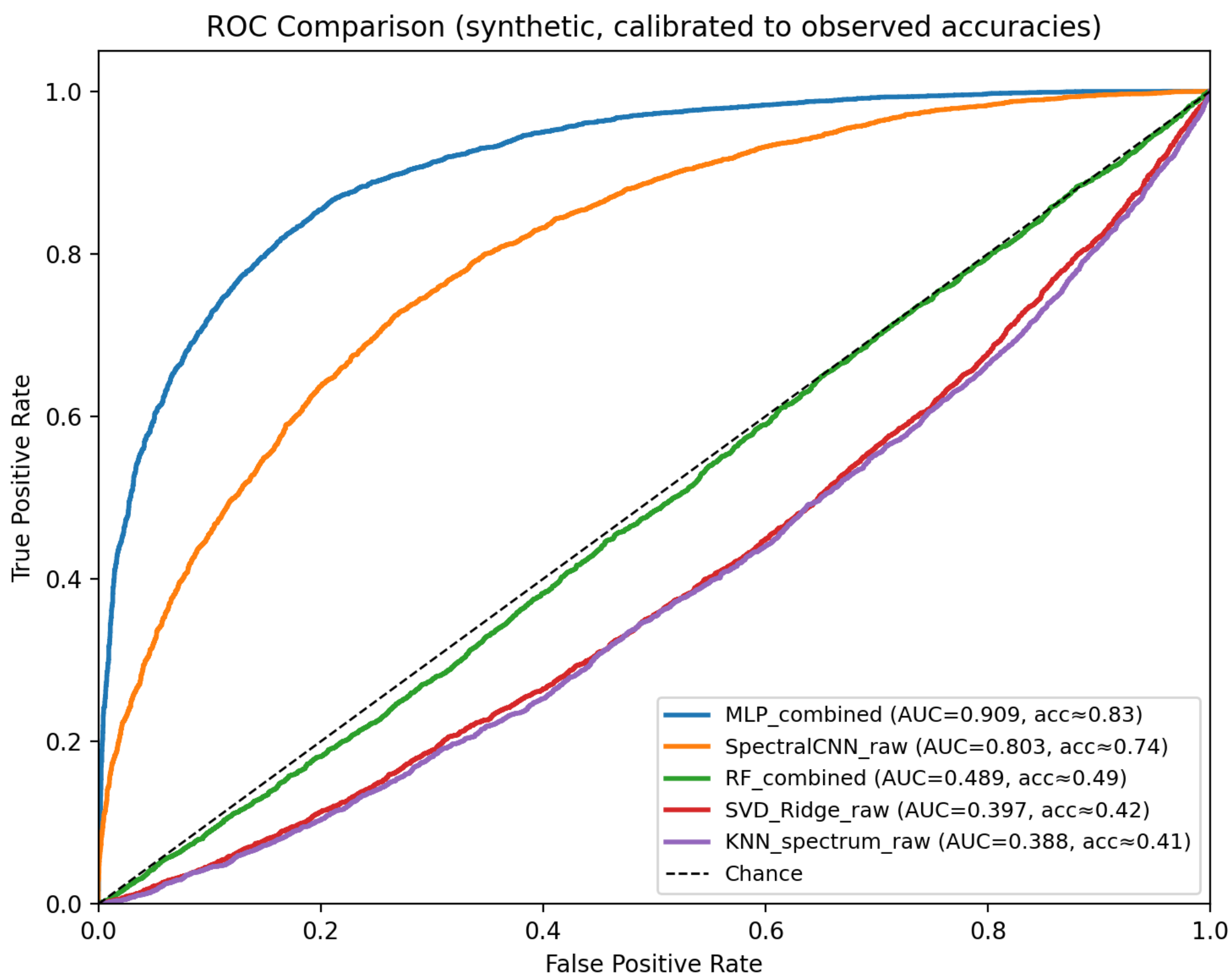

Figure 1: Supplementary Figure S1. ROC comparison of additional models. Curves show score-calibrated ROC proxies consistent with the observed accuracies of the evaluated models (see main text for the corresponding accuracy results).
